# Psychometric Validation of an Arabic‐Language Version of the Pictorial Empathy Test (PET) and the Single‐Item Trait Empathy Scale (SITES) in a Tunisian Young Adult Sample

**DOI:** 10.1002/mpr.70110

**Published:** 2026-09-25

**Authors:** Feten Fekih‐Romdhane, Ghada Amouchi, Amira Mohammed Ali, Majda Cheour, Souheil Hallit

**Affiliations:** ^1^ The Tunisian Center of Early Intervention in Psychosis Department of Psychiatry “Ibn Omrane” Razi Hospital Manouba Tunisia; ^2^ Faculty of Medicine of Tunis Tunis El Manar University Tunis Tunisia; ^3^ Department of Psychiatric Nursing and Mental Health Faculty of Nursing Alexandria University Alexandria Egypt; ^4^ School of Medicine and Medical Sciences Holy Spirit University of Kaslik Jounieh Lebanon; ^5^ Psychology Department College of Humanities Effat University Jeddah Saudi Arabia; ^6^ Applied Science Research Center Applied Science Private University Amman Jordan

**Keywords:** affective empathy, Arabic, empathy, psychometric properties, single‐item, trait empathy, validation

## Abstract

**Background:**

This study aims to examine the psychometric properties of an Arabic translation of two empathy measures, that is the Pictorial Empathy Test (PET) to assess affective empathic reactions and the Single Item Trait Empathy Scale (SITES) to assess empathy trait.

**Methods:**

This is a cross‐sectional study carried out during March‐May 2024 among 502 general population adults from Tunisia.

**Results:**

The one‐factor model of the PET scale showed excellent evidence of fit with good internal consistency (*α* = 0.86). Invariance across gender at the configural, metric, scalar and strict levels was established. Positive correlations were observed between PET and SITES scores. The SITES scores correlated significantly and positively with psychological distress. Both the SITES and the PET showed no significant correlations with self‐esteem scores.

**Conclusion:**

While the present validation study leaves further work to be completed, it provides to practitioners and researchers two brief, cost‐effective, valid and reliable measures of state (PET) and trait (SITES) empathy in the Arabic language.

## 1 Introduction

Empathy is essential at all ages, in everyday life and every part of society. Affective empathy refers to the ability to feel other people's emotional experiences indirectly, thus enabling us to comprehend their intentions and anticipate their behaviors (Baron‐Cohen 2004). During childhood, experiencing empathic feelings for others is crucial to emotional development (Bryant 1987) and contributes to the development of social morality (Agosta 2011). During adulthood, the ability to feel empathy allows effective interactions with people in social contexts. There is certain agreement that empathy includes two different components, cognitive and affective (or emotional) (Baron‐Cohen and Wheelwright 2004; Blair 2005), which are neurologically dissociable, underpinned by impairments in different areas of the brain (Shamay‐Tsoory et al. 2009; Bernhardt and Singer 2012). Cognitive empathy is being able to take the other's perspective (Davis 1983) or to understand the other's emotions (Jolliffe and Farrington 2006). Affective or emotional empathy is the ability to respond with an appropriate emotional reaction to the other's feelings or situation (Baron‐Cohen and Wheelwright 2004; Davis 1983). There are important interindividual differences in emotional reactions arising from contact with other people in the general population (Eisenberg and Strayer 1987); and such variations in empathic abilities were shown to affect, in turn, one's social life (Settoon and Mossholder 2002).

For instance, being able to feel empathy in a given situation, such as a person's suffering, can be impaired in traumatic brain injury (De Sousa et al. 2011), in schizophrenia (Bonfils et al. 2016), in high risk states of developing criminal behavior (van Zonneveld et al. 2017), and nonexistent or inappropriate in psychopathy (F. Ali et al. 2009). Affective empathic reactions have many positive effects on people's social life and interactions. For example, affective empathy is closely connected to helping behavior and being human in caring (e.g., psychotherapeutic) relationships (Kohut 1990). Possessing higher affective empathy is also associated with enhanced prosocial behavior (Sze et al. 2012), stronger intrinsic motivation for altruistic behavior (Feng et al. 2022), lower likelihood of engaging in traditional bullying (Utomo 2022) or substance using behavior (Winters et al. 2020), lower levels of aggression (Tampke et al. 2020), and a better quality of sleep (Guadagni et al. 2016). Empathy has also evidenced significant positive associations with enhanced self‐esteem (Findlay et al. 2006; L. Huang et al. 2019). Indeed, a number of previous studies indicated that self‐esteem acts as a positive predictor of empathy (Hongrui et al. 2016; Hui 2002). To explain this relationship, it has been suggested that empathy is empirically and theoretically connected to personality, and that thereby self‐esteem can influence empathy by its association with personality and one's value system (Iacobucci et al. 2013). Despite its positive social and behavioral attributes, affective empathy can also intensify empathic distress, potentially leading to the development of anxiety, depression, burnout, and other internalizing disorders (C. Huang et al. 2025). Indeed, the experience of empathy may lead to a confusion between self and other, and contribute, in turn, to psychological distress (Decety and Lamm 2011). For all these reasons, a large range of psychotherapeutic approaches highlight the role of empathy as an important component of treatment (Kaluzeviciute 2020). Given that affective empathy was consistently shown to be elementary to social behavior, and because its impairment was linked to a range of psychopathology and behavioral problems, its exploration and measurement in adults' populations represent a growing need for both clinicians and researchers. The following section expands on the different measurement tools currently available for the assessment of the (general and affective) empathy construct.

### 1.1 Measurement Instruments of Empathy

Empathy can be reflected through many psychological constructs, and measured using self‐administered tools (Kim and Lee 2010). A wide range of instruments have been developed and utilized to survey individuals' ability to empathize with others either in general or in specific contexts, such as empathy among physicians (Alcorta‐Garza et al. 2016), perceived relational empathy among patients in the consultation (Mercer et al. 2004), teachers' empathy for students (Warren 2015), empathy for the vicarious experience of pain (Giummarra et al. 2015), empathy experienced as anger on behalf of a victimized person, and ethnocultural empathy (Rasoal et al. 2011) (for systematic review and meta‐analysis, see (Lima and Osório 2021)). General empathy instruments available for use in the general population include the 60‐item Empathy Quotient (EQ) (Baron‐Cohen and Wheelwright 2004), the 28‐item Interpersonal Reactivity Index (Davis 1983), the Questionnaire of Cognitive and Affective Empathy (Reniers et al. 2011), the Questionary Measure of Emotional Empathy (Mehrabian and Epstein 1972), the Toronto Empathy Questionnaire (Spreng et al. 2009), the Empathy Assessment Index (Gerdes et al. 2011), the Affective and Cognitive Measure of Empathy (Vachon and Lynam 2016), and the 20‐item Basic Empathy Scale (Jolliffe and Farrington 2006) (which has been validated in Arabic in Tunisia (Dallagi‐Belkilani et al. 2024)). However, most of these measures showed limitations regarding factorial structure and convergent validity (Lima and Osório 2021), which is likely due to controversies and a lack of theoretical consensus on the multidimensional conceptualization of the empathy construct (Murphy et al. 2020). In addition, some of the existing measures were designed to and used in specific subgroups, such as the Basic Empathy Scale intended for assessing empathy in adolescents with antisocial tendencies (Jolliffe and Farrington 2006). Furthermore, one of the most recurring criticisms of empathy measures is that the construct being assessed is often unclear. For example, it was claimed that the EQ measures the ability to function socially rather than empathic tendencies of the individual (Lindeman et al. 2016); the Interpersonal Reactivity Index contains a “Personal Distress” and a “Fantasy” dimensions that are distinct from the concept of empathy (Baron‐Cohen and Wheelwright 2004); the Questionary Measure of Emotional Empathy appears to measure sympathetic reactions and emotional arousal by the environment in general rather than measuring empathy and other people's distress in particular (Jolliffe and Farrington 2004). As such, systematic reviews performed so far on measures of empathic ability both in specific populations (Jolliffe and Farrington 2004; Yu and Kirk 2009; Hong and Han 2020) and in the general population (Lima and Osório 2021) concluded that no instrument could be considered and recommended as the gold standard assessment method for empathy based on desirable validity and reliability indices. To address these gaps, (Lindeman et al. 2016) developed a brief test of affective empathic ability arousal, the Pictorial Empathy Test (PET), and (Konrath et al. 2018) developed the Single Item Trait Empathy Scale (SITES). The PET stands out from the existing self‐report measures that use text‐based items because it proposes a novel approach to conceptualize the empathy construct from an ecological perspective using photographs as emotional stimuli (Moya‐Albiol et al. 2010), while the SITES stands out because it consists of a single item (Lima and Osório 2021).

### 1.2 The PET

The PET was designed as a visual measure of affective empathy skills. It consists of 7 sequential photographs of people in vulnerable states (i.e., physical and/or emotional distress) and that are susceptible to elicit empathic emotions. Respondents are asked “How emotionally moving do you find the photograph?”, with answers ranging on a 5‐point Likert scale from 1 (not at all) to 5 (very much). Total PET scores can be obtained by calculating mean score of the responses, with higher scores reflecting greater affective empathy. The PET can be applied in different ways, as a web‐based questionnaire, as a paper‐and‐pencil test, or as part of an interview (Lindeman et al. 2016). As a photo‐based instrument, the PET has the potential advantage over other empathy scales of having stronger ecological validity in assessing affective empathy, given that facial expressions of emotions generate an “emotional resonance mechanism” in their watcher, and are thereby central to empathic responses (Balconi et al. 2011). The PET can also be appropriate and convenient‐to‐use when applied in clinical samples who have difficulty to express their answers in questionnaires (Lindeman et al. 2016). In addition, the PET is different from other measures, that are retrospective in nature, because it enables the immediate capture of respondents' emotional reactions and empathic feelings.

The PET was originally developed and validated in the Finnish language in three different samples of adults from the general population, where it demonstrated good internal reliability and 7‐month test‐retest reliability, adequate convergent validity, as well as a single‐latent‐factor structure (Lindeman et al. 2016). Other cross‐cultural adaptation studies have been carried out and reported equivalent psychometric characteristics to the original measure in different languages, countries and samples, including English (Monzel et al. 2023), Turkish (Şandor 2022) and Spanish (Baliyan et al. 2022). For instance, the psychometric characteristics of the English version of the PET were tested in a sample of 2232 adults recruited online, showing high reliability (Cronbach's *α* = 0.89) and confirming unidimensionality through exploratory factor analysis (Monzel et al. 2023). The Turkish version was administered to university students, in whom the PET had a Cronbach alpha coefficient of 0.78 as well as good convergent and concurrent validity as attested through significant positive correlations between PET scores and schizotypy Symptoms and EQ scores (Şandor 2022). The structure of the Spanish version of the PET was analyzed using exploratory then confirmatory factor analysis in a general population sample, in whom it yielded a single‐factor model with acceptable internal consistency (Cronbach's *α* = 0.77), high construct stability across gender groups, along with good discriminant, convergent, and nomological validity (Baliyan et al. 2022). As for the Arabic language, the psychometric properties of the PET have been recently investigated in a clinical sample of patients with schizophrenia from Tunisia, in whom it showed a unidimensional factor structure with excellent internal consistency (Cronbach's *α* = 0.93) (Kerbage et al. 2025). However, no previous validations of the Arabic PET exist to date in non‐clinical populations to the best of our knowledge.

### 1.3 The SITES

In contrast with the PET which assesses an emotional, situationally determined “state”, the SITES assesses empathy as a “trait” that is relatively stable across situations and over time. The SITES was proposed as a shorter and more specific alternative to the Interpersonal Reactivity Index (Davis 1983), which is composed of 28 items and theoretically designed to assess the same construct of trait empathy through different dimensions (i.e., Fantasy, Perspective‐taking, Empathic concern, and Personal distress). Respondents are asked to rate the extent to which the following statement describes them “I am an empathetic person”; the answer is rated from 1 (“Not very true of me”) to 5 (“Very true of me”) (Konrath et al. 2018). In seven studies and a large sample of adults (*N* = 5724), the SITES demonstrated good predictive validity, discriminant validity, and convergent validity with multiple dimensions of the Interpersonal Reactivity Index, and test‐retest reliability (Konrath et al. 2018). Overall, the SITES was shown to be a valid and reliable way to assess empathy when an efficient, rapid, but still accurate, method for data collection is demanded. In a sample of service employees from various industries, a study found significant positive associations between SITES and Empathy in Design Scale scores, supporting good convergent validity (Drouet et al. 2024). Another study also demonstrated positive correlations between trait empathy as measured using the SITES and social distancing among psychology undergraduates from the United States (Prachthauser 2021). Because its validity and reliability have been empirically established, the SITES may provide an efficient alternative to longer multi‐item instruments. As a single‐item measure, the SITES offers considerable practical advantages, including ease of administration and scoring, reduced survey fatigue and dropout rates, ease of integration in lengthy assessment batteries alongside other instruments, as well as greater feasibility and suitability in large‐scale and prospective studies with repeated assessment.

### 1.4 Rationale

The present study proposes to validate two empathy scales into the Arabic language, a pictorial, multi‐item measure assessing state‐level perception (the PET) and a verbal, single‐item measure assessing a broader trait‐level perception (the SITES). This approach is particularly valuable, as testing different measurement formats simultaneously allows to clarify whether these different approaches are psychometrically adequate and feasible in our target general population of Arabic‐speakers. Administering the PET and SITES together in this study offers an opportunity for examining convergent validity, as the two measures are expected to show a theoretically meaningful association. In sum, evaluating both instruments within the same sample was motivated by their complementary measurement characteristics and can establish a set of empathy measurements for future research in our context.

Validation studies in different linguistic and cultural contexts are crucial, as they allow researchers to generate and/or reinforce psychological theories that take into account the peculiarities of each cultural background (Gomes et al. 2018). Indeed, the vast majority of empathy measures, including the PET (for empathy state) and the SITES (for empathy trait), were developed and mostly used in Western societies of individualistic cultural orientation and it remains uncertain whether these scales also apply to individuals who grew up in the Arab collectivist cultural contexts. Additionally, there has been scant research on empathy in the Arab world. Arab nations are collectivist societies that place significant emphasis on group belonging, interdependence and social harmony (Hofstede 2001). In such cultural contexts, affective empathy focusing on emotional engagement with others tend to be fostered so that group unity can be maintain (Kitayama et al. 2000; Triandis 2001). Therefore, previous empirical evidence posited that individuals from collectivist societies tend to display greater empathic responses than those from individualist backgrounds due to high expectations in social roles and responsibilities put on them (Chopik et al. 2017; Schwartz et al. 2010). At the same time, however, more nuanced or even controversial findings have also emerged (Jami and Walker 2022). This mixed literature needs to be clarified and the way how empathy functions within different cultural environments should be further understood. Furthermore, the PET takes a few minutes and the SITES takes a few seconds to complete. As such, one potential benefit of these two scales over other empathy measures is that they are brief and could be easily completed by the respondent in a short amount of time and be easily interpreted by the evaluator, thus offering minimal respondent and administrative burden. The PET and SITES are therefore appropriate for use in Arab settings which often operate under severe human resources, time and monetary constraints.

To add to the literature, this study aims to examine the psychometric properties of an Arabic translation of the PET and SITES in a sample of Arabic‐speaking young adults from the general population of Tunisia. It is anticipated that the PET will show a unidimensional factor structure in both genders, good internal consistency reliability. It is also anticipated that good validity will be evidenced through adequate associations with relevant constructs (i.e., psychological distress and self‐esteem) consistently with previous studies (Findlay et al. 2006; L. Huang et al. 2019; C. Huang et al. 2025).

## 2 Methods

### 2.1 Participants and Procedure

This study has a cross‐sectional design and was carried out from March to May 2024. Eligible participants were adults from the general population of Tunisia, aged > 18 years, able to read and understand written Arabic, and with sufficient literacy to complete the online questionnaire. No exclusion criteria were applied, as the study aimed to evaluate the psychometric properties of the PET and SITES in a broad and heterogeneous sample with varying sociodemographic and individual characteristics that reflects the diversity of the general population, thereby providing a more comprehensive assessment of their applicability. Data was gathered using self‐report and web‐based questionnaires in the Arabic language. A soft copy of the questionnaire was created using Google Forms and distributed online through social media platforms. The snowball sampling method was adopted to recruit participants. Potential participants were approached by the research team, and were then asked to recruit other family members and friends. This process was continued, regardless of participants' gender. Before starting the survey, the study's information and general instructions were thoroughly explained. Participants' anonymity and confidentiality were preserved. The study protocol was approved by the ethics committee of Razi Hospital, Manouba, Tunisia (Reference: ECRPH‐2023‐0074).

Permission from the original authors, Professor Marjaana Lindeman (for the PET; (Lindeman et al. 2016)) and Professor Sara Konrath (for the SITES; (Konrath et al. 2018)), was obtained to translate and validate the scales. The forward and backward translation method was applied to the PET and SITES following international guidelines (Beaton et al. 2000). The first author supervised the translation and back‐translation process. The English version was translated to Arabic by a Tunisian translator who was completely unrelated to the study. Then, the Arabic version was translated back into English by another bilingual expert. The translated (Arabic) and original (English) versions were compared to detect any inconsistencies and eliminate them by a committee composed of the two translators and the research team who are all bilingual (Fekih‐Romdhane et al. 2022; Hallit et al. 2022). A pilot study on 30 persons was performed before the start of the official data collection to ensure that questions are well understood; no changes were deemed necessary based on the feedback from the pilot phase.

### 2.2 Measures

#### 2.2.1 Sociodemographic Information

The survey asked respondents to start by providing data regarding their age, gender (male, female), marital status (single, married, divorced, widowed), and family income categorized into < 500 Tunisian Dinar (TD), 500‐1000 TD, 1000‐2000 TD, 2000‐3000 TD and >◦3000 TD.

#### 2.2.2 The Depression, Anxiety and Stress Scales (DASS‐8)

The DASS‐8 is a short scale composed of 8 items which assess respondents' levels of psychological distress through three subscales: anxiety (3 items), depression (3 items), and stress (2 items). Each item is scored on a 4‐point Likert scale ranging from 0 (“does not apply to me”) to 3 (“always applies to me”). Higher scores reflect more severe psychological distress. The scale is validated in the Arabic language (A. M. Ali et al. 2024) (Current Cronbach's *α* = 0.84).

#### 2.2.3 The Arabic Single‐Item Self‐Esteem Scale (A‐SISE)

This is a single item measure (i.e., “I have high self‐esteem”) scored on a 5‐point Likert scale ranging from 1 (“not at all true of me”) to 5 (“very true of me”) (Brailovskaia and Margraf 2020). The Arabic validated version was used (Fekih‐Romdhane et al. 2023).

### 2.3 Analytic Strategy

There were no missing responses in the dataset. We conducted a confirmatory factor analysis (CFA) on the full sample using RStudio, with the “lavaan” and “SemTools” packages (Jorgensen et al. 2022; Rosseel et al. 2023). The analysis employed the Weighted Least Squares with Mean and Variance (WLSMV) estimator, which is recommended for ordinal data (Li 2016). Previous simulation studies suggest a sample size of approximately 150 is sufficient for a basic CFA model. Our sample was larger than this threshold. The root mean square error of approximation (RMSEA; ≤ 0.08), the standardized root mean square residual (SRMR; ≤ 0.08), the Tucker‐Lewis Index (TLI) and the comparative fit index (CFI) (≥ 0.90 for both) (Byrne 2013) were calculated to assess model fit. Multivariable normality was not verified as shown by Mardia's skewness = 713.85; *p* < 0.001 and Mardia's kurtosis = 16.53; *p* < 0.001. To examine gender invariance of PET scores, we conducted multi‐group CFA (Chen 2007) using the total sample. Measurement invariance was assessed at the configural, metric, and scalar levels (Vadenberg and Lance 2000) and verified if ΔCFI ≤ 0.010 and ΔRMSEA ≤ 0.015 or ΔSRMR ≤ 0.010 (Swami et al. 2022).

Regarding the SITES scale, we followed the recommendations of (Allen et al. 2022) who suggest that validating a single‐item scale should involve assessments of face validity, convergent validity and concurrent validity.

Composite reliability in both subsamples was assessed using McDonald's *ω* and Cronbach's *α*, with values greater than 0.70 reflecting adequate composite reliability for *α* (Dunn et al. 2014) and *ω* (McNeish 2018) values. The total PET and the SITES scores were considered normally distributed since their skewness and kurtosis values varied between −1 and +1 (Hair et al. 2010). To assess convergent and discriminant validity, we examined bivariate correlations between total PET and the SITES scores and those on the additional measures included in the survey using the Pearson correlation. Independent samples *t* test was used to compare two means. Based on Cohen (Cohen 1992), values ≤ 0.10 were considered weak, ∼ 0.30 were considered moderate, and ∼ 0.50 were considered strong correlations.

## 3 Results

Five hundred two participants filled the survey, with a mean age of 21.74 years (SD: 2.65) and 64.9% females. Other details can be found in Table 1.

**TABLE 1:** Sociodemographic characteristics of the participants (*n* = 502).

Gender	
Male	176 (35.1%)
Female	326 (64.9%)
Marital status	
Single	494 (98.4%)
Married	8 (1.6%)
Family income (in tunisian dinars)	
< 500	41 (8.3%)
500–1000	116 (23.4%)
1000–2000	191 (38.5%)
2000–3000	77 (15.5%)
> 3000	71 (14.3%)

### 3.1 Descriptive Statistics

Table 2 presents the descriptive statistics of the used scales. The SITES had a mean of 4.01 (SD = 0.93, range: 1–5), a median of 4.00, and the following score distribution: 0 = 0.2%, 1 = 1.0%, 2 = 2.6%, 3 = 27.1%, 4 = 32.3%, 5 = 36.9%.

**TABLE 2:** Descriptive statistics of all scores.

	Mean	SD	Min	Max	Skewness	Kurtosis
PET	3.73	0.92	1	5	−0.815	0.169
SITES	4.01	0.93	0	5	−0.657	0.208
Psychological distress	10.87	5.63	0	24	0.324	−0.701
Self‐esteem	4.00	0.93	1	5	−0.763	0.419

Abbreviations: PET = Pictorial Empathy Test; SITES = Single Item Empathy Scale.

### 3.2 Confirmatory Factor Analysis of the PET

**FIGURE 1:**
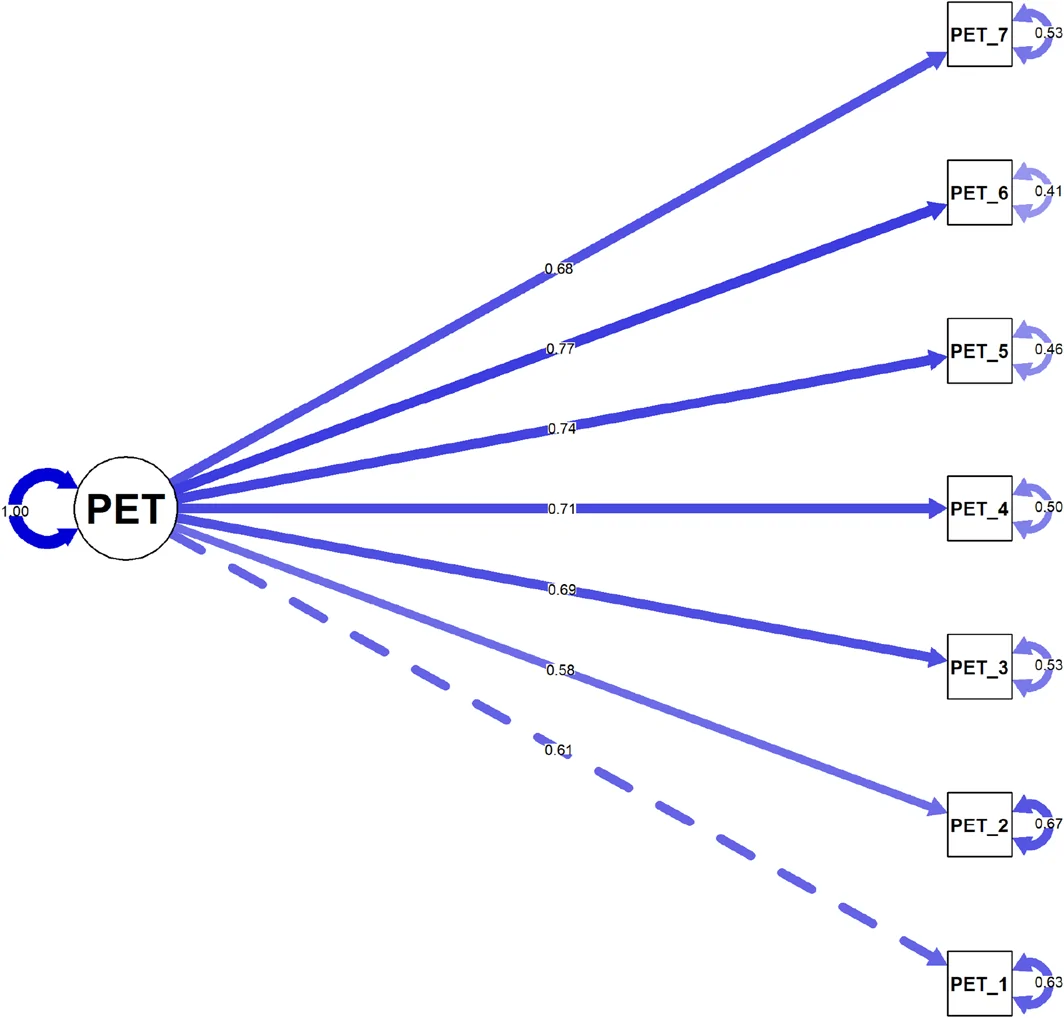
Standardized loading factors of the arabic version of the pictorial empathy test items.

The one‐factor model showed excellent evidence of fit (RMSEA = 0.051 (90% CI 0.028, 0.074), X^2^/df = 32.16/14 = 2.30, SRMR = 0.058, CFI = 0.989, and TLI = 0.984). The standardized estimates of factor loadings were all adequate (Figure 1). The internal consistency was good for the total score (*ω* = 0.85/*α* = 0.86).

### 3.3 Gender Invariance and Groups Comparison of the PET/SITES Scales

We were able to show the invariance across gender at the configural, metric, scalar and strict levels (Table 3). No significant difference was found between males and females in terms of PET scores (*M* = 26.16 (SD = 6.51) for males versus *M* = 26.13 (SD = 6.45) for females, t(500) = 0.06, *p* = 0.887, Cohen's *d* = 0.006) and SITES scores (*M* = 4.11 (SD = 0.90) for males versus *M* = 3.95 (SD = 0.94) for females, t(500) = 1.87, *p* = 0.077, Cohen's *d* = 0.175).

**TABLE 3:** Measurement invariance of the pictorial empathy test across gender.

Model	χ^2^	df	CFI	RMSEA	SRMR	Model comparison	ΔCFI	ΔRMSEA	ΔSRMR	Δχ^2^	Δdf	*p*
Configural	38.09	28	0.994	0.038	0.056							
Metric	48.04	34	0.992	0.041	0.063	Configural versus metric	0.002	0.003	0.007	9.95	6	0.127
Scalar	50.57	40	0.994	0.033	0.064	Metric versus scalar	0.002	0.008	0.001	2.53	6	0.865

Abbreviations: CFI = Comparative fit index; RMSEA = root mean square error of approximation; SRMR = Standardized root mean square residual.

### 3.4 Discriminant Validity

Both PET and SITES correlated positively and moderately with each other. PET scores did not correlate with psychological distress and self‐esteem, whereas higher SITES scores correlated weakly with higher psychological distress (Table 4).

**TABLE 4:** Correlation matrix.

	1	2	3	4
1. PET scores	1			
2. SITES	0.36***	1		
3. Psychological distress	0.04	0.21***	1	
4. Self‐esteem	0.02	−0.07	−0.19***	1
5. Age	0.03	−0.06	−0.004	0.06

*Note:* Numbers in the table indicate Pearson correlation coefficients (*r*).

Abbreviations: PET= Pictorial Empathy Test; SITES= Arabic Single Item Empathy Scale.

^***^ *p* < 0.001.

## 4 Discussion

This study offers an Arabic validated version of two empathy measures, the PET to assess affective empathic reactions and the SITES to assess general trait empathy among Arabic‐speaking populations. The psychometric examination indicated that the Arabic versions of the scales have good validity and reliability. In particular, CFA showed that the PET's unidimensionality was supported with an acceptable goodness‐of‐fit, and that good congruence of the factor structure of the SITES was supported across gender. Besides, the PET and the SITES exhibited satisfactory reliability coefficients. The validity of both scales was upheld via positive, weak correlations between SITES scores (trait empathy), but not PET scores (state empathy), and psychological distress.

Confirmatory Factor Analysis yielded a single factor structure of the PET. Our findings are consistent with both the original (Lindeman et al. 2016) and Spanish language (Baliyan et al. 2022) versions, showing that the loadings for all items exceeded the minimum recommended threshold of 0.30 (Brown 2015) and endorsing the earlier observation that the PET has a unidimensional factor structure with seven items. In addition, internal consistency reliability of the set of seven photographs was excellent in our Arabic‐speaking sample (McDonald's *ω* = 0.85 and Cronbach's *α* = 0.86), which is consistent with previous validation studies of the PET indicating high reliability in a sample of 91 Finnish‐speaking adults (*α* = 0.90) (Lindeman et al. 2016), in 79 Spanish‐speaking undergraduate psychology students (*α* = 0.77), in 580 Spanish‐speaking community adults (*α* = 0.86) (Baliyan et al. 2022), and in 374 Turkish‐speaking university students (*α* = 0.78) (Şandor 2022).

Positive correlations were observed between PET and SITES scores, which supports the convergence between the Arabic versions of the PET and the SITES and suggests that the single‐item measure is relevant and informative in assessing the trait empathy construct. The positive and moderate magnitude of the Pearson correlation coefficient between the two measures is explained by the fact that the PET assesses state empathy (Lindeman et al. 2016), whereas the SITES captures trait (or dispositional) empathy (Konrath et al. 2018). State empathy refers to affective empathic reactions at the moment they arise in response to the seven photographs (Lindeman et al. 2016), whereas trait empathy reflects the tendency for people to experience and imagine others' experiences and feelings, and is rather relatively stable across situations and over time (Konrath et al. 2018).

Furthermore, the SITES, but not the PET, scores correlated significantly and positively with psychological distress. These findings partially concur with growing evidence that great levels of affective empathy may result in overwhelming emotional states that contribute, in turn, to increased vulnerability to psychological distress (C. Huang et al. 2025). Given the well‐established fact that people with a great ability to feel empathy tend to be more vulnerable to psychological distress (Decety and Lamm 2011), the discrepancy observed in correlations with distress between the two scales is difficult to explain. Broadly in line with our results, (Bennik et al. 2019) found a positive relation between affective empathy and depressive symptoms when using the Quick Inventory of Depressive Symptomatology (QIDS, (Rush et al. 2003)), but not the DASS‐8. Authors explained their findings by the fact that DASS‐8 includes less physiological symptoms (Bennik et al. 2019). Therefore, the most plausible explanation could be that the PET and the SITES measure two distinct—yet related—facets of empathy, that are state empathy and trait empathy. The DASS‐8 is therefore less likely to correlate with instantaneous empathic emotional arousal as assessed using the PET, and significantly correlates in contrast with empathy when it is regarded as a stable personal trait. On another note, both the SITES and the PET showed no significant correlations with self‐esteem scores. An association between empathy and self‐esteem was predicted from a theoretical point of view, but there is currently limited and mixed evidence to support this idea (Sa et al. 2019). Previous research has shown either negative (Hanlong 2012), positive (L. Huang et al. 2019; Hongrui et al. 2016), or no more than a small association (Sa et al. 2019) between empathy and self‐esteem. Cultural influences may explain in part the lack of association between self‐esteem and empathy in our sample, as both constructs are culturally‐dependent (Hamamura 2017; Hollan 2017) and can be related to each other differently across cultures and contexts. Previous cross‐cultural research has shown that individuals from collectivist societies tend to report lower self‐esteem and higher affective empathy levels than those from individualist societies (Schmitt and Allik 2005; Errasti Pérez et al. 2018). Indeed, collectivists seem to be generally more emotionally involved in their relationship with others (Hofstede 1984), and this emotional involvement does not seem to relate to self‐esteem. Rather, there is evidence that perceived esteem (i.e., perceptions of how positively one is viewed by others, not by the self), independent of self‐worth, is particularly important to social relationships (Hermann et al. 2008).

Comparison of empathy levels between genders showed no significant differences between males and females, both when using the PET to assess state empathy or the SITES to assess trait empathy. There is a “widespread stereotypical belief” that males and females differ in their ability to be empathic (Löffler and Greitemeyer 2023), and that females are characterized by being more empathetic than males (Christov‐Moore et al. 2014). However, the scientific validity of this assumption is questionable, as empirical evidence showed inconsistent findings. Several studies concur with our result and have reported no significant gender difference (Kim and Lee 2010; Konrath et al. 2018; Lamm et al. 2011), some studies found that females tended to score higher in empathy compared to males (Reniers et al. 2011; Derntl et al. 2010), whereas other research has even noted higher male competence (Lennon et al. 1986). These controversial findings across studies may be explained by the fact that societal and cultural effects on gender differences are most pronounced in explicit evaluations in which individuals are requested to produce an empathy‐related behavior (Gleichgerrcht and Decety 2013; Eisenberg and Lennon 1983). In addition, it is important to note that the affective empathy phenomenon involves a range of influencing variables than gender, such as individual differences in skills for recognizing others' emotions (Besel and Yuille 2010).

### 4.1 Study Limitations

Some limitations should be acknowledged. First, data was gathered in one time point, which means that test‐retest reliability and predictive validity could not be investigated in the context of the present study, and need to be considered in future studies. Testing temporal stability is particularly critical for the SITES scale in order to establish whether this empathy trait measure yields consistent scores over time. In addition, participants consisted of Arabic‐speaking individuals from a single country and culture (Tunisia), were highly educated, unmarried, and young, which may limit the generalizability of our findings to adults from other Arab nationalities and cultural backgrounds. Future studies should therefore explore the applicability of the PET and SITES in other Arabic‐speaking countries. The representativeness and generalizability of our study's findings to larger populations can also be hindered by the online and snowball sampling method adopted, which mostly attracted young people with a university level of education. Moreover, the possibility of non‐response bias and social desirability effects cannot be ruled out, especially given the self‐report and socially sensitive nature of the constructs being measured.

### 4.2 Implications and Future Perspectives

The present results suggest that the Arabic‐language versions of the PET and the SITES are valid and reliable measures of affective empathy and trait empathy, respectively. Due to their brevity, simplicity and easiness of use for practitioners and researchers, both the PET and the SITES can be adopted in settings where there is financial or time strains, and could be suitable methods for large‐scale surveys, longitudinal studies, and pre‐screening assessments. The two measures may help reduce administration burden, improve data quality, and enable to simultaneously investigate wide masses of respondents where necessary. In particular, as a photograph‐based test, the PET could be the best and most helpful alternative to assess affective empathy reactions in the moment of administration in patients who may have problems in identifying and verbalizing their empathic feelings with a text‐based questionnaire. Moreover, through a single item that can be completed in seconds, the SITES helps measure the specific aspect of how caring and empathic people are in nature. It is therefore recommended for use in different situations or settings where longer scales measuring the same construct are not appropriate (due to their length, cost, amount of time and burden generated) or do not suit the outcome of interest (trait empathy specifically vs. dimensions such as “Fantasy” and “Personal Distress”). At the same time, we recognize that this study's limitations leave several areas open for future research. As our study fully relied on self‐report measures, further studies using behavioral measurements of empathy are warranted to enhance accuracy of our findings. In addition, other psychometric testing studies establishing test–retest reliability and inter‐rater reliability are still needed to confirm the stability of the PET and the SITES over time and across different rates. Finally, the two measures were validated in a non‐clinical adult sample, and their validation in clinical populations (such as patients with schizophrenia or other mental health conditions) remain to be undertaken.

## 5 Conclusion

While the present validation study leaves further work to be completed, it provides to practitioners and researchers two measures of empathy in the Arabic language. The two scales are brief, cost‐effective, well‐accepted, and can be easily understood by participants. We thus recommend their use in future clinical and research practices, in particular in settings where resources and time can be limited. Future work enrolling multiple groups across Arab countries and longitudinal designs would help readers situate the study in a wider agenda. Making the PET and SITES available in the Arabic language will hopefully benefit patients and stimulate future research in this area in the Arab region, and further advance knowledge about empathy, its correlates and its cross‐cultural variation.

## Author Contributions

**Feten Fekih‐Romdhane:** conceptualization (lead), writing – original draft (lead), investigation (lead), methodology (lead), project administration (lead), supervision (lead), writing – review and editing (equal). **Ghada Amouchi:** data curation (lead), writing – review and editing (equal). **Amira Mohammed Ali:** writing – review and editing (equal), validation (supporting). **Majda Cheour:** writing – review and editing (equal), validation (supporting). **Souheil Hallit:** software (lead), formal analysis (lead), Validation (lead), investigation (supporting), methodology (supporting), writing – review and editing (equal).

## Funding

The authors have nothing to report.

## Ethics Statement

Each participant provided a voluntary digital informed consent before beginning the survey. The research protocol was approved by the ethics committee of the Razi psychiatric hospital, Manouba, Tunisia (Ref: ECRPH‐2023‐0074). The study was performed following the standards for medical research involving human subjects recommended by the Declaration of Helsinki for human research.

## Consent

The authors have nothing to report.

## Conflicts of Interest

The authors declare no conflicts of interest.

## Data Availability

The datasets generated and/or analyzed during the current study are publicly available: https://osf.io/g28cu/.

## References

[mpr70110-bib-0001] Agosta, L. 2011. Empathy and Sympathy in Ethics.

[mpr70110-bib-0002] Alcorta‐Garza, A. , M. San‐Martín , R. Delgado‐Bolton , J. Soler‐González , H. Roig , and L. Vivanco . 2016. “Cross‐Validation of the Spanish HP‐Version of the Jefferson Scale of Empathy Confirmed With Some Cross‐Cultural Differences.” Frontiers in Psychology 7: 1002. 10.3389/fpsyg.2016.01002. PMC4940391 27462282

[mpr70110-bib-0003] Ali, A. M. , A. A. Alkhamees , S. Hallit , T. N. Al‐Dwaikat , H. Khatatbeh , and S. A. Al‐Dossary . 2024. “The Depression Anxiety Stress Scale 8: Investigating Its Cutoff Scores in Relevance to Loneliness and Burnout Among Dementia Family Caregivers.” Scientific Reports 14, no. 1: 13075. 10.1038/s41598-024-60127-1. PMC11156668 38844485

[mpr70110-bib-0004] Ali, F. , I. S. Amorim , and T. Chamorro‐Premuzic . 2009. “Empathy Deficits and Trait Emotional Intelligence in Psychopathy and Machiavellianism.” Personality and Individual Differences 47, no. 7: 758–762. 10.1016/j.paid.2009.06.016.

[mpr70110-bib-0005] Allen, M. S. , D. Iliescu , and S. Greiff . 2022. Single Item Measures in Psychological Science. Hogrefe Publishing.

[mpr70110-bib-0006] Balconi, M. , A. Bortolotti , and L. Gonzaga . 2011. “Emotional Face Recognition, EMG Response, and Medial Prefrontal Activity in Empathic Behaviour.” Neuroscience Research 71, no. 3: 251–259. 10.1016/j.neures.2011.07.1833. 21840350

[mpr70110-bib-0007] Baliyan, S. , J. M. Cimadevilla , A. Bustillos , et al. 2022. “Cultural Adaptation, Validation, and Psychometric Description of the Pictorial Empathy Test (PET) in the Spanish Population.” European Journal of Psychological Assessment.

[mpr70110-bib-0008] Baron‐Cohen, S. 2004. Questione Di cervello: La differenza essenziale tra uomini e donne: Mondadori.

[mpr70110-bib-0009] Baron‐Cohen, S. , and S. Wheelwright . 2004. “The Empathy Quotient: An Investigation of Adults With Asperger Syndrome or High Functioning Autism, and Normal Sex Differences.” Journal of Autism and Developmental Disorders 34, no. 2: 163–175. 10.1023/b:jadd.0000022607.19833.00. 15162935

[mpr70110-bib-0010] Beaton, D. E. , C. Bombardier , F. Guillemin , and M. B. Ferraz . 2000. “Guidelines for the Process of Cross‐Cultural Adaptation of Self‐Report Measures.” Spine 25, no. 24: 3186–3191. 10.1097/00007632-200012150-00014. 11124735

[mpr70110-bib-0011] Bennik, E. C. , B. F. Jeronimus , and M. aan het Rot . 2019. “The Relation Between Empathy and Depressive Symptoms in a Dutch Population Sample.” Journal of Affective Disorders 242: 48–51. 10.1016/j.jad.2018.08.008. 30173062

[mpr70110-bib-0012] Bernhardt, B. C. , and T. Singer . 2012. “The Neural Basis of Empathy.” Annual Review of Neuroscience 35: 1–23. 10.1146/annurev-neuro-062111-150536. 22715878

[mpr70110-bib-0013] Besel, L. D. , and J. C. Yuille . 2010. “Individual Differences in Empathy: The Role of Facial Expression Recognition.” Personality and Individual Differences 49, no. 2: 107–112. 10.1016/j.paid.2010.03.013.

[mpr70110-bib-0014] Blair, R. J. R. 2005. “Responding to the Emotions of Others: Dissociating Forms of Empathy Through the Study of Typical and Psychiatric Populations.” Consciousness and Cognition 14, no. 4: 698–718. 10.1016/j.concog.2005.06.004. 16157488

[mpr70110-bib-0015] Bonfils, K. A. , P. H. Lysaker , K. S. Minor , and M. P. Salyers . 2016. “Affective Empathy in Schizophrenia: A Meta‐Analysis.” Schizophrenia Research 175, no. 1: 109–117. 10.1016/j.schres.2016.03.037. 27094715

[mpr70110-bib-0016] Brailovskaia, J. , and J. Margraf . 2020. “How to Measure Self‐Esteem With One Item? Validation of the German Single‐Item Self‐Esteem Scale (G‐SISE).” Current Psychology: A Journal for Diverse Perspectives on Diverse Psychological Issues 39, no. 6: 2192–2202. 10.1007/s12144-018-9911-x.

[mpr70110-bib-0017] Brown, T. A. 2015. Confirmatory Factor Analysis for Applied Research. Guilford publications.

[mpr70110-bib-0018] Bryant, B. K. 1987. Mental Health, Temperament, Family, and Friends: Perspectives on Children's Empathy and Social Perspective Taking.

[mpr70110-bib-0019] Byrne, B. M. 2013. Structural Equation Modeling with Mplus: Basic Concepts, Applications, and Programming. routledge.

[mpr70110-bib-0020] Chen, F. F. 2007. “Sensitivity of Goodness of Fit Indexes to Lack of Measurement Invariance.” Structural Equation Modeling: A Multidisciplinary Journal 14, no. 3: 464–504. 10.1080/10705510701301834.

[mpr70110-bib-0021] Chopik, W. J. , E. O’Brien , and S. H. Konrath . 2017. “Differences in Empathic Concern and Perspective Taking Across 63 Countries.” Journal of Cross‐Cultural Psychology 48, no. 1: 23–38. 10.1177/0022022116673910.

[mpr70110-bib-0022] Christov‐Moore, L. , E. A. Simpson , G. Coudé , K. Grigaityte , M. Iacoboni , and P. F. Ferrari . 2014. “Empathy: Gender Effects in Brain and Behavior.” Neuroscience & Biobehavioral Reviews 46: 604–627. 10.1016/j.neubiorev.2014.09.001. PMC5110041 25236781

[mpr70110-bib-0023] Cohen, J. 1992. “Quantitative Methods in Psychology: A Power Primer.” In Psychological Bulletin. 10.1037//0033-2909.112.1.155 19565683

[mpr70110-bib-0024] Dallagi‐Belkilani, M. , M. Olivier , and C. Besche‐Richard . 2024. “Validation of the Basic Empathy Scale in an Arabic‐Speaking Population: The BES‐Ar.” L'Encéphale 50, no. 2: 149–153. 10.1016/j.encep.2023.04.002. 37088580

[mpr70110-bib-0025] Davis, M. H. 1983. “Measuring Individual Differences in Empathy: Evidence for a Multidimensional Approach.” Journal of Personality and Social Psychology 44, no. 1: 113–126. 10.1037/0022-3514.44.1.113.

[mpr70110-bib-0026] Decety, J. , and C. Lamm . 2011. “15 Empathy Versus Personal Distress: Recent Evidence From Social Neuroscience.” Social Neuroscience of Empathy: 199.

[mpr70110-bib-0027] Derntl, B. , A. Finkelmeyer , S. Eickhoff , et al. 2010. “Multidimensional Assessment of Empathic Abilities: Neural Correlates and Gender Differences.” Psychoneuroendocrinology 35, no. 1: 67–82. 10.1016/j.psyneuen.2009.10.006. 19914001

[mpr70110-bib-0028] De Sousa, A. , S. McDonald , J. Rushby , S. Li , A. Dimoska , and C. James . 2011. “Understanding Deficits in Empathy After Traumatic Brain Injury: The Role of Affective Responsivity.” Cortex 47, no. 5: 526–535. 10.1016/j.cortex.2010.02.004. 20334857

[mpr70110-bib-0029] Drouet, L. , K. Bongard‐Blanchy , and C. Lallemand . 2024. “Development of the Empathy in Design Scale: Measuring Employees’ Empathy Toward Users in Service Design.” Interacting with Computers 36, no. 5: 313–334. 10.1093/iwc/iwae019.

[mpr70110-bib-0030] Dunn, T. J. , T. Baguley , and V. Brunsden . 2014. “From Alpha to Omega: A Practical Solution to the Pervasive Problem of Internal Consistency Estimation.” British Journal of Psychology 105, no. 3: 399–412. 10.1111/bjop.12046. 24844115

[mpr70110-bib-0031] Eisenberg, N. , and R. Lennon . 1983. “Sex Differences in Empathy and Related Capacities.” Psychological Bulletin 94, no. 1: 100–131. 10.1037/0033-2909.94.1.100.

[mpr70110-bib-0032] Eisenberg, N. , and J. Strayer . 1987. Critical Issues in the Study of Empathy.

[mpr70110-bib-0033] Errasti Pérez, J. M. , I. Amigo Vázquez , J. M. Villadangos Fernández , and J. Morís Fernández . 2018. “Differences Between Individualist and Collectivist Cultures in Emotional Facebook Usage: Relationship With Empathy, Self‐Esteem, and Narcissism.” Psicothema 30, no. 4: 376–381. 10.7334/psicothema2018.101. 30353837

[mpr70110-bib-0034] Fekih‐Romdhane, F. , Z. Bitar , R. Rogoza , et al. 2023. “Validity and Reliability of the Arabic Version of the Self‐Report Single‐Item Self‐Esteem Scale (A‐SISE).” BMC Psychiatry 23, no. 1: 351. 10.1186/s12888-023-04865-y. PMC10201777 37217890

[mpr70110-bib-0035] Fekih‐Romdhane F. , M. Fawaz , R. Hallit , T. Sawma , S. Obeid , and S. Hallit , 2022. “Psychometric Properties of an Arabic Translation of the Multidimensional Social Support Scale (MSPSS) in a Community Sample of Lebanese Adults.”. 10.1186/s12888-023-04937-z PMC10265861 37316897

[mpr70110-bib-0036] Feng, H. , Y. Zeng , and E. Lu . 2022. “Brain‐Inspired Affective Empathy Computational Model and Its Application on Altruistic Rescue Task.” Frontiers in Computational Neuroscience 16: 784967. 10.3389/fncom.2022.784967. PMC9341284 35923916

[mpr70110-bib-0037] Findlay, L. C. , A. Girardi , and R. J. Coplan . 2006. “Links Between Empathy, Social Behavior, and Social Understanding in Early Childhood.” Early Childhood Research Quarterly 21, no. 3: 347–359. 10.1016/j.ecresq.2006.07.009.

[mpr70110-bib-0038] Gerdes, K. E. , C. A. Lietz , and E. A. Segal . 2011. “Measuring Empathy in the 21st Century: Development of an Empathy Index Rooted in Social Cognitive Neuroscience and Social Justice.” Social Work Research 35, no. 2: 83–93. 10.1093/swr/35.2.83.

[mpr70110-bib-0039] Giummarra, M. J. , B. Fitzgibbon , N. Georgiou‐Karistianis , et al. 2015. “Affective, Sensory and Empathic Sharing of Another's Pain: The E Mpathy for P Ain S Cale.” European Journal of Pain 19, no. 6: 807–816. 10.1002/ejp.607. 25380353

[mpr70110-bib-0040] Gleichgerrcht, E. , and J. Decety . 2013. “Empathy in Clinical Practice: How Individual Dispositions, Gender, and Experience Moderate Empathic Concern, Burnout, and Emotional Distress in Physicians.” PLoS One 8, no. 4: e61526. 10.1371/journal.pone.0061526. PMC3631218 23620760

[mpr70110-bib-0041] Gomes, L. B. , C. N. Bossardi , S. D. A. Bolze , et al. 2018. “Cross‐Cultural Research in Developmental Psychology: Theoretical and Methodological Considerations.” Arquivos Brasileiros de Psicologia 70, no. 1: 260–275. https://pepsic.scielo.org/scielo.php?script=sci_arttext&pid=S1809‐52672018000100018.

[mpr70110-bib-0042] Guadagni, V. , F. Burles , S. Valera , et al. 2016. “The Relationship Between Quality of Sleep and Emotional Empathy.” Journal of Psychophysiology.

[mpr70110-bib-0043] Hair J. F. Jr ., W. C. Black , B. J. Babin , and R. E. Anderson . 2010. “Multivariate Data Analysis.” In: Multivariate Data Analysis: 785.

[mpr70110-bib-0044] Hallit S. , Z. Bitar , R. Rogoza , S. Obeid . 2022. “Validation of the Arabic Version of the Freiburg Mindfulness Inventory (FMI‐Ar) Among a Sample of Lebanese University Students.”.

[mpr70110-bib-0045] Hamamura T. 2017. “Cultural Differences in Self‐Esteem.” In: Encyclopedia of Personality and Individual Differences, edited by Zeigler‐Hill V. , Shackelford T. K. : 1–3. Springer International Publishing.

[mpr70110-bib-0046] Hanlong, L. 2012. The Characteristics of Medical Students’ Empathy and Relevant Factors. Tianjin Medical University.

[mpr70110-bib-0047] Hermann, A. D. , G. M. Lucas , and J. Friedrich . 2008. “Individual Differences in Perceived Esteem Across Cultures.” Self and Identity 7, no. 2: 151–167. 10.1080/15298860701319044.

[mpr70110-bib-0048] Hofstede, G. 1984. Culture's Consequences: International Differences in Work‐Related Values, Vol. 5. Sage.

[mpr70110-bib-0049] Hofstede, G. 2001. Culture's Consequences: Comparing Values, Behaviors, Institutions and Organizations Across Nations. Sage publications.

[mpr70110-bib-0050] Hollan D. 2017. “Empathy Across Cultures.” In: The Routledge Handbook of Philosophy of Empathy: 341–352. Routledge.

[mpr70110-bib-0051] Hong, H. , and A. Han . 2020. “A Systematic Review on Empathy Measurement Tools for Care Professionals.” Educational Gerontology 46, no. 2: 72–83. 10.1080/03601277.2020.1712058.

[mpr70110-bib-0052] Hongrui, Z. , Z. Hui , L. Xiaofan , L. Fenghua , and J. Shuyao . 2016. “The Current Status and Correlation of Empathy Ability and Self‐Esteem Level Among Nursing Students.” Nursing Journal of Chinese People’s Liberation Army 33: 1–4.

[mpr70110-bib-0053] Huang, C. , Z. Wu , S. Sha , et al. 2025. “The Dark Side of Empathy: The Role of Excessive Affective Empathy in Mental Health Disorders.” Biological Psychiatry 98, no. 5: 404–415. 10.1016/j.biopsych.2024.12.020. 39793690

[mpr70110-bib-0054] Huang, L. , J. Thai , Y. Zhong , H. Peng , J. Koran , and X.‐D. Zhao . 2019. “The Positive Association Between Empathy and Self‐Esteem in Chinese Medical Students: A Multi‐Institutional Study.” Frontiers in Psychology 10: 1921. 10.3389/fpsyg.2019.01921. PMC6712570 31496978

[mpr70110-bib-0055] Hui, J. 2002. Research on the Mechanism of Empathy in Moral Behavior and Value of Moral Education. Nanjing Normal University.

[mpr70110-bib-0056] Iacobucci, T. A. , B. J. Daly , D. Lindell , and M. Q. Griffin . 2013. “Professional Values, Self‐Esteem, and Ethical Confidence of Baccalaureate Nursing Students.” Nursing Ethics 20, no. 4: 479–490. 10.1177/0969733012458608. 23166146

[mpr70110-bib-0057] Jami, P. Y. , and D. I. Walker . 2022. “Exploring Situational Empathy and Intergroup Empathy Bias Among People With Two Opposing Cultural Norms: Collectivism and Individualism.” International Journal of Intercultural Relations 91: 282–296. 10.1016/j.ijintrel.2022.11.002.

[mpr70110-bib-0058] Jolliffe, D. , and D. P. Farrington . 2004. “Empathy and Offending: A Systematic Review and Meta‐Analysis.” Aggression and Violent Behavior 9, no. 5: 441–476. 10.1016/j.avb.2003.03.001.

[mpr70110-bib-0059] Jolliffe, D. , and D. P. Farrington . 2006. “Development and Validation of the Basic Empathy Scale.” Journal of Adolescence 29, no. 4: 589–611. 10.1016/j.adolescence.2005.08.010. 16198409

[mpr70110-bib-0102] Jorgensen, T. D. , S. Pornprasertmanit , A. M. Schoemann , and Y. Rosseel . 2022. “SemTools: Useful Tools for Structural Equation Modeling (0.5‐6).” [Computer Software]. https://cran.r‐project.org/web/packages/semTools/index.html.

[mpr70110-bib-0061] Kaluzeviciute, G. 2020. “The Role of Empathy in Psychoanalytic Psychotherapy: A Historical Exploration.” Cogent Psychology 7, no. 1: 1748792. 10.1080/23311908.2020.1748792.

[mpr70110-bib-0062] Kerbage, G. , C. Akkari , N. Hachem , et al. 2025. “Psychometric Properties of the Arabic Version of the Pictorial Empathy Test for Assessing Affective Empathic Reactions in Patients With Schizophrenia.” Healthcare 13, no. 16: 2022. 10.3390/healthcare13162022. PMC12385803 40868637

[mpr70110-bib-0063] Kim, J. , and S. J. Lee . 2010. “Reliability and Validity of the Korean Version of the Empathy Quotient Scale.” Psychiatry Investigation 7, no. 1: 24. 10.4306/pi.2010.7.1.24. PMC2848769 20396429

[mpr70110-bib-0064] Kitayama, S. , H. R. Markus , and M. Kurokawa . 2000. “Culture, Emotion, and Well‐Being: Good Feelings in Japan and the United States.” Cognition & Emotion 14, no. 1: 93–124. 10.1080/026999300379003.

[mpr70110-bib-0065] Kohut, H. 1990. “The Role of Empathy in Psychoanalytic Cure.” In Classics in Psychoanalytic Techniques, 463–473.

[mpr70110-bib-0066] Konrath, S. , B. P. Meier , and B. J. Bushman . 2018. “Development and Validation of the Single Item Trait Empathy Scale (SITES).” Journal of Research in Personality 73: 111–122. 10.1016/j.jrp.2017.11.009. PMC5839508 29527069

[mpr70110-bib-0067] Lamm, C. , J. Decety , and T. Singer . 2011. “Meta‐Analytic Evidence for Common and Distinct Neural Networks Associated With Directly Experienced Pain and Empathy for Pain.” NeuroImage 54, no. 3: 2492–2502. 10.1016/j.neuroimage.2010.10.014. 20946964

[mpr70110-bib-0068] Lennon, R. , N. Eisenberg , and J. Carroll . 1986. “The Relation Between Nonverbal Indices of Empathy and Preschoolers' Prosocial Behavior.” Journal of Applied Developmental Psychology 7, no. 3: 219–224. 10.1016/0193-3973(86)90030-4.

[mpr70110-bib-0069] Li, C. H. 2016. “Confirmatory Factor Analysis With Ordinal Data: Comparing Robust Maximum Likelihood and Diagonally Weighted Least Squares.” Behavior Research Methods 48, no. 3: 936–949. 10.3758/s13428-015-0619-7. 26174714

[mpr70110-bib-0070] Lima, F. Fd , and F. d. L. Osório . 2021. “Osório FdL: Empathy: Assessment Instruments and Psychometric Quality–A Systematic Literature Review With a Meta‐Analysis of the Past Ten Years.” Frontiers in Psychology 12: 781346. 10.3389/fpsyg.2021.781346. PMC8653810 34899531

[mpr70110-bib-0071] Lindeman, M. , I. Koirikivi , and J. Lipsanen . 2016. “Pictorial Empathy Test (PET).” European Journal of Psychological Assessment.

[mpr70110-bib-0072] Löffler, C. S. , and T. Greitemeyer . 2023. “Are Women the More Empathetic Gender? the Effects of Gender Role Expectations.” Current Psychology 42, no. 1: 220–231. 10.1007/s12144-020-01260-8.

[mpr70110-bib-0073] McNeish, D. 2018. “Thanks Coefficient Alpha, We'll Take it From Here.” Psychological Methods 23, no. 3: 412–433. 10.1037/met0000144. 28557467

[mpr70110-bib-0074] Mehrabian, A. , and N. Epstein . 1972. “A Measure of Emotional Empathy.” Journal of Personality. 10.1111/j.1467-6494.1972.tb00078.x 4642390

[mpr70110-bib-0075] Mercer, S. W. , M. Maxwell , D. Heaney , and G. C. Watt . 2004. “The Consultation and Relational Empathy (CARE) Measure: Development and Preliminary Validation and Reliability of an Empathy‐Based Consultation Process Measure.” Family Practice 21, no. 6: 699–705. 10.1093/fampra/cmh621. 15528286

[mpr70110-bib-0076] Monzel, M. , K. Keidel , and M. Reuter . 2023. “Is it Really Empathy? The Potentially Confounding Role of Mental Imagery in Self‐Reports of Empathy.” Journal of Research in Personality 103: 104354. 10.1016/j.jrp.2023.104354.

[mpr70110-bib-0077] Moya‐Albiol, L. , N. Herrero , and M. C. Bernal . 2010. “The Neural Bases of Empathy.” Revista de Neurologia 50, no. 2: 89–100. 10.33588/rn.5002.2009111. 20112217

[mpr70110-bib-0078] Murphy, B. A. , T. H. Costello , A. L. Watts , Y. F. Cheong , J. M. Berg , and S. O. Lilienfeld . 2020. “Strengths and Weaknesses of Two Empathy Measures: A Comparison of the Measurement Precision, Construct Validity, and Incremental Validity of Two Multidimensional Indices.” Assessment 27, no. 2: 246–260. 10.1177/1073191118777636. 29847994

[mpr70110-bib-0079] Prachthauser, M. 2021. Construction of the Social Distance Scale and the Relationship Between Trait Empathy and Social Distancing.

[mpr70110-bib-0080] Rasoal, C. , T. Jungert , S. Hau , and G. Andersson . 2011. “Development of a Swedish Version of the Scale of Ethnocultural Empathy.” Psychology 2, no. 6: 568–573. 10.4236/psych.2011.26087.

[mpr70110-bib-0081] Reniers, R. L. , R. Corcoran , R. Drake , N. M. Shryane , and B. A. Völlm . 2011. “The QCAE: A Questionnaire of Cognitive and Affective Empathy.” Journal of Personality Assessment 93, no. 1: 84–95. 10.1080/00223891.2010.528484. 21184334

[mpr70110-bib-0082] Rosseel, Y. , D. Oberski , J. Byrnes , et al. 2023. “Lavaan: Latent Variable Analysis (0.6‐16).” [Computer Software]. https://cran.r‐project.org/web/packages/lavaan/index.html.

[mpr70110-bib-0083] Rush, A. J. , M. H. Trivedi , H. M. Ibrahim , et al. 2003. “The 16‐Item Quick Inventory of Depressive Symptomatology (QIDS), Clinician Rating (QIDS‐C), and Self‐Report (QIDS‐SR): A Psychometric Evaluation in Patients With Chronic Major Depression.” Biological Psychiatry 54, no. 5: 573–583. 10.1016/s0006-3223(02)01866-8. 12946886

[mpr70110-bib-0084] Sa, B. , N. Ojeh , M. A. A. Majumder , et al. 2019. “The Relationship Between Self‐Esteem, Emotional Intelligence, and Empathy Among Students From Six Health Professional Programs.” Teaching and Learning in Medicine 31, no. 5: 536–543. 10.1080/10401334.2019.1607741. 31075996

[mpr70110-bib-0085] Şandor, S. 2022. “Mediator Effects of Cognitive and Affective Empathy on the Relationship Between Schizotypal Symptoms and Social Anxiety/Avoidance Levels.” Neuropsychiatric Investigation 60, no. 3: 64–71. 10.5152/neuropsychiatricinvest.2022.21004.

[mpr70110-bib-0086] Schmitt, D. P. , and J. Allik . 2005. “Simultaneous Administration of the Rosenberg Self‐Esteem Scale in 53 Nations: Exploring the Universal and Culture‐Specific Features of Global Self‐Esteem.” Journal of Personality and Social Psychology 89, no. 4: 623–642. 10.1037/0022-3514.89.4.623. 16287423

[mpr70110-bib-0087] Schwartz, S. J. , J. B. Unger , B. L. Zamboanga , and J. Szapocznik . 2010. “Rethinking the Concept of Acculturation: Implications for Theory and Research.” American Psychologist 65, no. 4: 237–251. 10.1037/a0019330. PMC3700543 20455618

[mpr70110-bib-0088] Settoon, R. P. , and K. W. Mossholder . 2002. “Relationship Quality and Relationship Context as Antecedents of Person‐and Task‐Focused Interpersonal Citizenship Behavior.” Journal of Applied Psychology 87, no. 2: 255–267. 10.1037/0021-9010.87.2.255. 12002954

[mpr70110-bib-0089] Shamay‐Tsoory, S. G. , J. Aharon‐Peretz , and D. Perry . 2009. “Two Systems for Empathy: A Double Dissociation Between Emotional and Cognitive Empathy in Inferior Frontal Gyrus Versus Ventromedial Prefrontal Lesions.” Brain 132, no. 3: 617–627. 10.1093/brain/awn279. 18971202

[mpr70110-bib-0090] Spreng, R. N. , M. C. McKinnon , R. A. Mar , and B. Levine . 2009. “The Toronto Empathy Questionnaire: Scale Development and Initial Validation of a Factor‐Analytic Solution to Multiple Empathy Measures.” Journal of Personality Assessment 91, no. 1: 62–71. 10.1080/00223890802484381. PMC2775495 19085285

[mpr70110-bib-0091] Swami, V. , J. Todd , V. Azzi , et al. 2022. “Psychometric Properties of an Arabic Translation of the Functionality Appreciation Scale (FAS) in Lebanese Adults.” Body Image 42: 361–369. 10.1016/j.bodyim.2022.07.008. 35926365

[mpr70110-bib-0092] Sze, J. A. , A. Gyurak , M. S. Goodkind , and R. W. Levenson . 2012. “Greater Emotional Empathy and Prosocial Behavior in Late Life.” Emotion 12, no. 5: 1129–1140. 10.1037/a0025011. PMC3766763 21859198

[mpr70110-bib-0093] Tampke, E. C. , P. J. Fite , and J. L. Cooley . 2020. “Bidirectional Associations Between Affective Empathy and Proactive and Reactive Aggression.” Aggressive Behavior 46, no. 4: 317–326. 10.1002/ab.21891. PMC8075037 32227484

[mpr70110-bib-0094] Triandis, H. C. 2001. “Individualism‐Collectivism and Personality.” Journal of Personality 69, no. 6: 907–924. 10.1111/1467-6494.696169. 11767823

[mpr70110-bib-0095] Utomo, K. D. M. 2022. “Investigations of Cyber Bullying and Traditional Bullying in Adolescents on the Roles of Cognitive Empathy, Affective Empathy, and Age.” International Journal of Instruction 15, no. 2: 937–950. 10.29333/iji.2022.15251a.

[mpr70110-bib-0096] Vachon, D. D. , and D. R. Lynam . 2016. “Fixing the Problem With Empathy: Development and Validation of the Affective and Cognitive Measure of Empathy.” Assessment 23, no. 2: 135–149. 10.1177/1073191114567941. 25612628

[mpr70110-bib-0097] Vadenberg, R. , and C. Lance . 2000. “A Review and Synthesis of the Measurement in Variance Literature: Suggestions, Practices, and Recommendations for Organizational Research.” Organizational Research Methods 3: 4–70. 10.1177/109442810031002.

[mpr70110-bib-0098] van Zonneveld, L. , E. Platje , L. de Sonneville , S. Van Goozen , and H. Swaab . 2017. “Affective Empathy, Cognitive Empathy and Social Attention in Children at High Risk of Criminal Behaviour.” Journal of Child Psychology and Psychiatry 58, no. 8: 913–921. 10.1111/jcpp.12724. 28397960

[mpr70110-bib-0099] Warren, C. A. 2015. “Scale of Teacher Empathy for African American Males (S‐TEAAM): Measuring Teacher Conceptions and the Application of Empathy in Multicultural Classroom Settings.” Journal of Negro Education 84, no. 2: 154–174. 10.7709/jnegroeducation.84.2.0154.

[mpr70110-bib-0100] Winters, D. E. , W. Wu , and S. Fukui . 2020. “Longitudinal Effects of Cognitive and Affective Empathy on Adolescent Substance Use.” Substance Use & Misuse 55, no. 6: 983–989. 10.1080/10826084.2020.1717537. 32067568

[mpr70110-bib-0101] Yu, J. , and M. Kirk . 2009. “Evaluation of Empathy Measurement Tools in Nursing: Systematic Review.” Journal of Advanced Nursing 65, no. 9: 1790–1806. 10.1111/j.1365-2648.2009.05071.x. 19694842

